# Adverse Perinatal and Early Life Outcomes following 15q11.2 CNV Diagnosis

**DOI:** 10.3390/genes12101480

**Published:** 2021-09-23

**Authors:** Fu-Chieh Chu, Steven W. Shaw, Chien-Hong Lee, Liang-Ming Lo, Jenn-Jeih Hsu, Tai-Ho Hung

**Affiliations:** 1 Department of Obstetrics and Gynecology, Taipei Chang Gung Memorial Hospital, Taipei 114, Taiwan, China; dadajay@hotmail.com (F.-C.C.); doctor.obsgyn@gmail.com (S.W.S.); lmlo@cgmh.org.tw (L.-M.L.); jjhsu@ms6.hinet.net (J.-J.H.); 2 College of Medicine, Chang Gung University, Taoyuan 330, Taiwan, China; 3 Department of Laboratory Medicine, Chang Gung Memorial Hospital, Linkou Medical Center, Taoyuan 333, Taiwan, China; li5592@cgmh.org.tw; 4 Department of Obstetrics and Gynecology, Keelung Chang Gung Memorial Hospital, Keelung 204, Taiwan, China

**Keywords:** 15q11.2 BP1–BP2 microdeletion, Burnside–Butler syndrome, 15q11.2 microduplication, *TUBGCP5*, *CYFIP1*, *NIPA1*, *NIPA2*

## Abstract

The copy number variation (CNV) of 15q11.2, an emerging and common condition observed during prenatal counseling, is encompassed by four highly conserved and non-imprinted genes—*TUBGCP5*, *CYFIP1*, *NIPA1*, and *NIPA2*—which are reportedly related to developmental delays or general behavioral problems. We retrospectively analyzed 1337 samples from genetic amniocentesis for fetal CNV using microarray-based comparative genomic hybridization analysis between January 2014 and December 2019. 15q11.2 CNV showed a prevalence of 1.5% (21/1337). Separately, 0.7% was noted for 15q11.2 BP1–BP2 microdeletion and 0.8% for 15q11.2 microduplication. Compared to the normal array group, the 15q11.2 BP1–BP2 microdeletion group had more cases of neonatal intensive care unit transfer, an Apgar score of <7 at 1 min, and neonatal death. Additionally, the group was symptomatic with developmental delays and had more infantile deaths related to congenital heart disease (CHD). Our study makes a novel contribution to the literature by exploring the differences in the adverse perinatal outcomes and early life conditions between the 15q11.2 CNV and normal array groups. Parent-origin gender-based differences may help in the prognosis of the fetal phenotype; development levels should be followed up in the long term and echocardiography should be offered prenatally and postnatally for the prevention of a delayed diagnosis of CHD.

## 1. Introduction

The copy number variation (CNV) of 15q11.2 BP1–BP2 is an emerging and common situation associated with pregnant women during prenatal obstetrician counseling. With an estimated prevalence ranging from 0.57 to 1.27%, 15q11.2 BP1–BP2 microdeletion was first described by Murthy et al. in 2007 and named Burnside–Butler syndrome [1,2,3,4,5]. 15q11.2 BP1–BP2 microdeletion is considered a recurrent susceptibility CNV with increased risk of attention-deficit/hyperactivity disorder, autism spectrum disorder, epilepsy, and congenital heart disease (CHD) [2,6]. The region of 15q11.2 is located between the proximal 15q breakpoints BP1 and BP2 and includes four highly conserved and non-imprinted protein coding OMIM genes: *TUBGCP5, CYFIP1, NIPA1,* and *NIPA2*. Each of these four genes has been reported to be expressed in the central nervous system, associated with neurodevelopmental disorder, and cause clinical pathogenic phenotype variations. However, the gene interaction and pathways remain unclear. Rafi and Butler (2020) noted, via in silico analyses, that a protein network interaction model found associations between these four coding genes and neurodevelopmental disorders [6]. However, further in vitro research is still required in the future to recognize the gene pathways.

According to a review studying 200 patients [2], the symptoms of 15q11.2 BP1–BP2 microdeletion can be classified into five main categories: (1) developmental (73%), speech (67%), and motor delays (42%); (2) facial dysmorphism (46%); (3) writing (60%) and reading (57%) difficulties, memory problems (60%), and verbal intelligence quotient (IQ) scores less than 75 (50%); (4) general behavioral problems (55%); and (5) abnormal brain imaging (43%). Nevertheless, 15q11.2 CNV is inherited from unaffected parents in approximately 80% of children [1,2,7,8]. It is challenging to predict the neonatal phenotype based on low penetrance (10.4%) and a variable phenotype expressivity [9,10]. Therefore, the 15q11.2 BP1–BP2 microdeletion is considered as a recurrent susceptibility copy number variant in public databases (e.g., DECIPHER, ClinGen, and ClinVar). However, the 15q11.2 BP1–BP2 microdeletion has been found to be the leading cytogenetic finding of autism spectrum disorder [11,12]. As most of the current research is focused on psychological problems and developmental conditions, we aimed to retrospectively review the results of amniocentesis and focus on the adverse perinatal outcomes and early life conditions following 15q11.2 BP1–BP2 CNV. We also compared the early life outcomes between the 15q11.2 BP1–BP2 CNV groups and the normal array results group. We demonstrated the fetal genotype–phenotype correlation to update the current database and to improve the accuracy in prenatal obstetrician counseling.

## 2. Materials and Methods

### 2.1. Study Population

**Figure 1:**
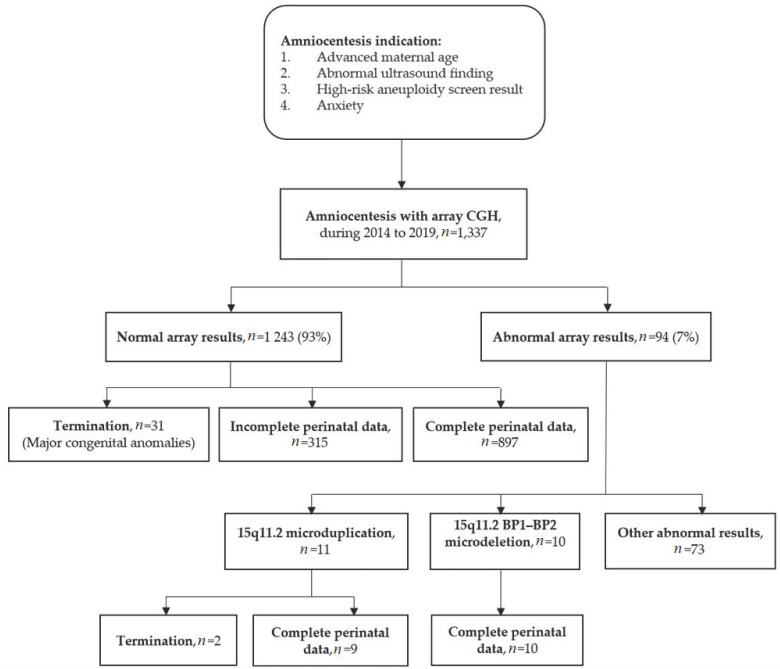
Flow diagram and the common indications for amniocentesis.

A total of 1,337 prenatal amniocentesis samples were obtained for fetal karyotyping and concomitant CNV using microarray-based comparative genomic hybridization analysis (array CGH) between January 2014 and December 2019 at the Department of Obstetrics and Gynecology, Taipei and Linkuo Branches of Chang Gung Memorial Hospital, Taiwan. A flow diagram and the common indications for amniocentesis are presented in Figure 1. We retrospectively analyzed the results of array CGH and identified 1243 samples with normal array results (1243/1337, 93%) and 94 samples with abnormal array results (94/1337, 7%). Of the 1243 samples with normal array results, 31 cases terminated their pregnancy owing to major congenital anomalies, while 315 cases had incomplete perinatal data; thus, only 897 cases were included in this study with complete perinatal and infantile outcomes. Meanwhile, of the 94 samples with abnormal array results, 21 cases had 15q11.2 BP1–BP2 CNV. Karyotype and array CGH did not identify other chromosomal aneuploidies or alterations in these 21 cases. Of these cases, 10 samples had 15q11.2 BP1–BP2 microdeletion and 11 samples had 15q11.2 microduplication. Two cases with microduplication chose to terminate their pregnancy during the second trimester. Thus, data of only 10 cases with 15q11.2 BP1–BP2 microdeletion and nine cases with 15q11.2 microduplication were ultimately analyzed for perinatal and early life outcomes in this study. We compared maternal demographics, pregnancy characteristics, and perinatal and early life outcomes between (1) 15q11.2 BP1–BP2 microdeletion, (2) 15q11.2 microduplication, and (3) normal array results.

### 2.2. Definitions of Adverse Perinatal and Infantile Outcomes

We examined the following maternal demographics and pregnancy characteristics: maternal age at delivery; advanced maternal age (defined as >34 years of age); primiparity; gestational age (estimated based on the first day of the mother’s last normal menstrual period or by ultrasound dating if the date was uncertain); follow-up period (the period immediately after birth and last clinical visit); inheritance pattern (classified as paternal origin, maternal origin, or de novo); symptoms of inherited parents with 15q11.2 BP1–BP2 CNV, including developmental delay, psychological disease, or congenital anomalies identified by a dysmorphologist.

The following adverse perinatal and infantile outcomes were studied: cesarean section (CS); postpartum hemorrhage (blood loss >1000 mL after CS or >500 mL from vaginal delivery); maternal comorbidity (systemic comorbidities such as hypertension, cardiovascular disease, diabetes, renal disease, infectious disease, thyroid disease, anemia, mental disorders, and others; localized comorbidities such as placental disease, chorioamnionitis, amniotic sac disorders, cervical incompetence, and structural abnormalities); preterm birth (delivery <37 weeks of gestation); low birth weight (birth weight <2500 g); macrosomia (birth weight >4000 g); transfer to a neonatal intensive care unit (NICU); Apgar score <7 at 1 and 5 min; neonatal death (within 1 month after birth); infantile death (within 1 year after birth); development levels, diagnosed and screened based on the Comprehensive Developmental Inventory for Infants and Toddlers [13], which was designed for infants and children aged 3–71 months in Taiwan, including growth delay (children’s height < mean height minus two standard deviations (SDs) for the same gender and population), speech and language delay (delayed speech according to the age-related milestones), and motor delay (slow development of fine- or gross-motor abilities); facial dysmorphism; general behavioral problems (attention-deficit/hyperactivity disorder (ADHD), obsessive–compulsive disorder (OCD), emotional behavioral disorder, or anxiety); CHD, excluding patent foramen ovale and patent ductus arteriosus (PDA); and abnormal brain imaging with magnetic resonance or ultrasonography.

### 2.3. Array CGH Analysis

The SurePrint G3 Human CGH Microarray Kit, 60,000 probes (Agilent Technologies, Santa Clara, CA, USA) platform was used for the identification of chromosomal abnormalities in this study. DNA was extracted using a QIAamp DNA Blood Mini Kit (Qiagen, Hilden, Germany) and labeled via a SureTag DNA Labeling Kit (Agilent Technology) with Cy3-dUTP according to the manufacturers’ instructions. The sex-matched reference human genomic DNA was labeled with Cy5-dUTP. Finally, the results were scanned using a SureScan Microarray scanner (Agilent Technology) and analyzed with Feature Extraction Software v11.5 (Agilent Technology), using the Human Genome Browser (hg19).

### 2.4. Statistical Analysis

Statistical analyses were performed using MedCalc for Windows, version 15.12.0 (MedCalc Software, Ostend, Belgium). The continuous variables are presented as mean ± SD, whereas the categorical variables were calculated as number and rate (%). Comparisons between these three groups were calculated via one-way ANOVA or *χ*^2^ tests, as appropriate. A *p*-value <0.05 was considered significant.

## 3. Results

From the retrospective analyses, the prevalence of 15q11.2 BP1–BP2 CNV was approximately 1.5% (21/1337), while 15q11.2 BP1–BP2 microdeletion was 0.7% (10/1337) and 15q11.2 microduplication was 0.8% (11/1337). The population with the indication of abnormal prenatal ultrasound finding that received amniocentesis through array CGH during 2014 to 2019 in Chang Gung Memorial Hospital was about 8.6% (115/1,337). Among the group of abnormal prenatal ultrasound finding, the calculated prevalence of 15q11.2 BP1–BP2 microdeletion was 2.6% (3/115) and that of 15q11.2 microduplication was 0.9% (1/115).

The clinical data of those children with 15q11.2 BP1–BP2 CNV are presented in Table 1 and Table 2. The maternal characteristics and mean follow-up time of the study participants are presented in Table 3. No significant differences were observed between these three groups. The inheritance pattern and symptoms of the inherited parents are listed in Table 4. In the 15q11.2 BP1–BP2 microdeletion group, 7 of the 10 patients had their inheritance pattern analyzed; all of the patients had inherited the 15q11.2 BP1–BP2 microdeletion, with 57.1% of paternal origin and 42.9% of maternal origin. Additionally, parents carrying the 15q11.2 BP1–BP2 microdeletion tended to have more symptomatic congenital anomalies compared to parents carrying the 15q11.2 microduplication, including three paternal carriers with CHD and one maternal carrier with short stature. Alternatively, in the microduplication group, 18.2% of the patients were de novo, with 36.4% of paternal origin and 45.5% of maternal origin—none of the parents had symptomatic congenital anomalies.

Next, we investigated the perinatal outcomes, as shown in Table 5. There were no significant differences in the maternal outcomes. However, compared to the normal array group, the 15q11.2 BP1–BP2 microdeletion group had more cases of NICU transfer, Apgar scores <7 at 1 min, and neonatal deaths. Three neonates were sent to the NICU due to hypoxemia related to CHD, and Case 10 died within one month.

The early life outcomes are listed in Table 6. Compared to the normal array and microduplication groups, children in the 15q11.2 BP1–BP2 microdeletion group tended to be more symptomatic (70%), especially with developmental delay (50%), including 30% with growth delays, 20% with speech delays, and 10% with motor delays. Additionally, both of the 15q11.2 CNV groups had a tendency of have more facial dysmorphism, including Case 5 with 15q11.2 BP1–BP2 microdeletion, who had a prominent occiput pattern, protruding eyes, low-set ears, and small oral and narrow palate arches, and Case 12 with microduplication, who had hypertelorism. Most importantly, the 15q11.2 BP1–BP2 microdeletion group had more infantile deaths related to CHD compared to the normal array group. Although there was also no significant difference in the CHD between these three groups, we found three CHDs (30%) in the 15q11.2 BP1–BP2 microdeletion group. Case 2 was diagnosed with a secundum-type atrial septal defect of 4.9 mm, PDA of 2.6 mm, mild pulmonary stenosis, and persistent left superior vena cava (PLSVC) but survived and is currently four years old. Case 5 was diagnosed with mid-aortic syndrome with a secundum-type atrial septal defect of 3.6 mm, PDA of 3.9 mm, moderate pulmonary stenosis, persistent left superior vena cava (PLSVC), corpus callosum hypogenesis, and facial dysmorphism and died three months later. Lastly, Case 10 was diagnosed with a secundum-type atrial septal defect of 13 mm with severe aortic stenosis, moderate pulmonary stenosis, and PDA of 5.5 mm and died within one month. Infantile death was found in 20% of cases (2/10) due to the sequential change of CHD. From abnormal brain imaging, five cases in the microdeletion group were ordered to have postnatal brain ultrasonography or MRI; Case 5 with multiple anomalies was also diagnosed with corpus callosum hypogenesis with bilateral lateral ventriculomegaly. In the microduplication group, Case 18 was diagnosed with bilateral subdural effusion and bilateral lateral ventriculomegaly.

## 4. Discussion

The 15q11.2 region has four highly conserved and non-imprinted OMIM genes in the BP1–BP2 region: *TUBGCP5, CYFIP1, NIPA1*, and *NIPA2*. According to the DECIPHER database, *CYFIP1* (cytoplasmic fragile X mental retardation interacting protein 1) and *NIPA2* (non-imprinted in Prader–Willi/Angelman syndrome 2) are pathogenic genes and are associated with neuronal cytoskeletal remodeling. *CYFIP1* interacts with fragile X mental retardation protein (FMRP; absent FMRP causes fragile X syndrome); reduced *CYFIP1* in human neural progenitors results in the dysregulation of the schizophrenia and epilepsy gene networks [14]. *TUBGCP5* (tubulin γ complex associated protein 5), a less-intolerant gene and highly expressed in the brain, is associated with OCD and ADHD [15,16,17]. *NIPA1* (non-imprinted in Prader–Willi/Angelman syndrome 1) and *NIPA2* (non-imprinted in Prader–Willi/Angelman syndrome 2) are both magnesium ion transporters and are highly expressed in brain tissue; moreover, *NIPA1* is less intolerant. An impaired magnesium ion transporter decreases the intracellular magnesium concentration in neurons, enhances N-methyl-D-aspartate receptor, and ultimately impacts neuron excitability and brain function [18]. Mutations in *NIPA1* are associated with autosomal dominant hereditary spastic paraplegia 6 and postural disturbance [19], while mutations in *NIPA2* are associated with childhood absence epilepsy [18]. In addition, although *TUBGCP5* and *NIPA1* have been found to be expressed in the fetal heart, their roles in heart development remain undetermined [20]. A recent report noted—via in silico analyses—that these four coding genes are associated with 10 overlapping neurodevelopmental disorders [6]. The protein network interaction model suggests all four genes to be individually associated with Prader–Willi syndrome, autism spectrum disorder (ASD), schizophrenia, epilepsy, and Down syndrome, with *CYFIP1* as the only gene associated with a developmental disorder.

15q11.2 BP1–BP2 microdeletion is more symptomatic than 15q11.2 microduplication. The reduced expression of *CYFIP1, NIPA1, NIPA2*, and *TUBGCP5* has an impact on growth, language, speech, and psychomotor development and CHD. In our microduplication group data, 22.2% (2/9) of the symptomatic infants had either hypertelorism or bilateral subdural effusion. There seems to be no obvious effect on the maternal and perinatal outcomes compared to the normal array group. However, in the 15q11.2 BP1–BP2 microdeletion group, more infantile deaths (20%) were noted. In addition, approximately 70% (7/10) of the symptomatic children had developmental delays (50%)—especially growth delays (30%), speech delays (20%), or motor delays (10%). Although no striking differences were observed during prenatal echocardiography between these three groups, three neonates were diagnosed with CHD then transferred to the NICU due to hypoxemia, and ultimately two neonates died.

In Simon et al.’s unselected cohort study of 500,000 individuals via UK Biobank, the prevalence of 15q11.2 microdeletion was shown to be approximately 0.38%. 15q11.2 microdeletion also increases the risk of neuropsychiatric phenotypes (OR = 1.84, 95% CI 1.23–2.75) and congenital cardiovascular malformation (OR = 1.73, 95% CI = 1.08–2.75) [21]. As our mean follow-up time was 25 months, the general behavioral problems, IQ scores, and learning difficulties were difficult to identify and examine. However, in our 15q11.2 BP1–BP2 microdeletion group, CHD occurred in approximately 30% of patients, and the disease of all was of paternal origin. According to Butler’s review, with regard to the parent-origin effect, gender-based differences were observed [22]. Maternal origin deletion was associated with macrocephaly and autism spectrum disorder, while paternal origin deletion was associated with CHD.

In etiologies of CHD, while 35% of the patients were considered to be associated with genetic factors, 65% remain unknown. The major frequent pathogenic CNVs in CHD are 22q11.2 deletion (DiGeorge syndrome), 7q11.23 deletion (Williams–Beuren syndrome), and 17p11 deletion (Smith–Magenis syndrome) [23]. 15q11.2 BP1–BP2 microdeletion is also associated with the increased risk of sporadic CHD (OR = 8.2; *p* = 0.02) [20]. However, the gene interaction and pathway need further evaluation.

Our study had the following limitations:

(1) A small sample size, thus leading to an insufficient study population; in Taiwan, although amniocentesis for array examination is suggested if there is a clinical indication, the prevalence of prenatal array examination in Taiwan is approximately 6% [24]. Thus, more cases are required to update the current database and demonstrate infantile outcomes.
(2) The mean follow-up time was only 25 months; age is the most widely recognized modifier for phenotype penetrance, as increased age is frequently associated with increased penetrance rates. Moreover, because the general behavioral problems, IQ scores, or learning difficulties were difficult to identify in children over two years of age, a follow-up evaluation during the period of elementary school could be considered.
(3) Further detailed assessments for pedigree may help us to illustrate the incomplete penetrance for dominant traits and genotype–phenotype relationships.
(4) The causes of incomplete penetrance cannot be explained in this condition; the incidence of CHD seems higher (30%) in our study compared to previous studies (2–17%) [4,25]. The increased prevalence of CHD could be related to selection bias, as the common indication for amniocentesis in Taiwan is abnormal fetal ultrasound findings.
(5) Further DNA sequencing is needed to exclude other pathogenic gene mutations. Based on genetic heterogeneity, these patients may have other genetic congenital point mutations that can cause CHD, which cannot be analyzed via CGH array. Moreover, the effect of environmental modifications or genetic co-factors for incomplete penetrance and the variable expressivity of 15q11.2 BP1–BP2 microdeletion syndrome were difficult to identify in our study.

## 5. Conclusions

CNVs of 15q11.2 are emerging and commonly observed during prenatal genetic counseling. Our study demonstrates that the incidence of prenatally diagnosed 15q11.2 CNV was 1.5%; the prevalence of 15q11.2 BP1–BP2 microdeletion was 0.7% and of 15q11.2 microduplication was 0.8%. Among the group of abnormal prenatal ultrasound finding, the calculated prevalence of 15q11.2 BP1–BP2 microdeletion was 2.6% and that of 15q11.2 microduplication was 0.9%. Microduplication of 15q11.2 seems to be a likely benign variant and does not affect perinatal and infantile outcomes. However, 15q11.2 BP1–BP2 microdeletion syndrome has a variable phenotype expressivity and low penetrance of approximately 10%, and 42.9% of these are inherited from unaffected parents. In clinical obstetrician counseling, parent-origin gender-based differences may help in the prognosis for the fetal phenotypes. Development levels should be followed up in the long term, and serial prenatal and postnatal fetal echocardiography follow-ups are required to prevent delayed diagnosis of CHD, as most of our cases did not show obvious abnormal cardiac findings during mid-second trimester fetal echocardiography. Delivery at tertiary care centers is also suggested for possible NICU care and cardiologist intervention to prevent infantile death. In the future, more in vitro research is required to understand the genetic causation, as well as the gene interaction and pathway of 15q11.2 BP1–BP2 copy number variants.

## Figures and Tables

**Table 1:** Clinical characteristics of children diagnosed with 15q11.2 BP1–BP2 microdeletion.

Patient Number	Karyotype	Array	Inherited Origin	Symptoms of Parents	Infantile Outcome	Follow-Up Period (Month)
1	46, XY	15q11.2(22,765,628_23,217,514) × 1	Paternal	No	Normal	67
2	46, XX	15q11.2(22,765,628_23,217,514) × 1	Paternal	CHD	CHD (secundum-type atrial septal defect, PDA, PS, and PLSVC)	50
3	46, XY	15q11.2(22,765,628_23,217,514) × 1	NA	No	Growth delay	43
4	46, XX	15q11.2(22,765,628_23,217,514) × 1	NA	No	Speech delay and motor delay	40
5	46, XX	15q11.2(22,765,628_23,146,132) × 1	NA	No	Mid-aortic syndrome CHD (secundum-type atrial septal defect, PDA, PS, and PLSVC) Facial dysmorphism Corpus callosum hypogenesis	Expired within 3 months
6	46, XX	15q11.2(22,770,421_23,276,605) × 1	Paternal	CHD	Normal	31
7	46, XX	15q11.2(22,765,628_23,217,514) × 1	Maternal	Short stature Depression	Growth delay	30
8	46, XY	15q11.2(22,765,628_23,217,514) × 1	Maternal	No	Speech delay	22
9	46, XX	15q11.2(22,765,628_23,217,514) × 1	Maternal	No	Normal	6
10	46, XX	15q11.2(22,765,628_23,217,514) × 1	Paternal	CHD	CHD (secundum-type atrial septal defect, PDA, PS, AS, and PLSVC)	Expired within 1 month

Abbreviations: CHD, congenital heart disease; ASD, atrial septal defect; PDA, patent ductus arteriosus; PS, pulmonary stenosis; PLSVC, persistent left superior vena cava; AS, arterial stenosis; NA, not available.

**Table 2:** Clinical characteristics of children diagnosed with 15q11.2 microduplication.

Patient Number	Karyotype	Array	Inherited Origin	Symptoms of Parents	Infantile Outcome	Follow-Up Period (Months)
11	46, XY	15q11.2(22,765,628_23,300,287) × 3	De novo	No	Elective termination at the second trimester	NA
12	46, XY	15q11.2(22,765,628_23,086,303) × 3	Maternal	No	Hypertelorism	63
13	46, XX	15q11.2(22,765,628_23,300,287) × 3	Maternal	No	Normal	56
14	46, XY	15q11.2(22,770,421_23,625,785) × 4	De novo	No	Normal	49
15	46, XX	15q11.2(23,967,663_24,478,240) × 3	Maternal	No	Normal	36
16	46, XX	15q11.2(22,765,628_23,300,287) × 3	Paternal	No	Elective termination at the second trimester	NA
17	46, XY	15q11.2(22,765,628_23,300,287) × 3	Maternal	No	Normal	21
18	46, XX	15q11.2(22,765,628_23,300,287) × 3	Paternal	No	Bilateral ventriculomegaly and subdural effusion	15
19	46, XX	15q11.2(22,765,628_23,300,287) × 3	Paternal	No	Normal	9
20	46, XX	15q11.2(22,765,628_23,300,287) × 3	Paternal	No	Normal	6
21	46, XY	15q11.2(22,765,628_23,300,287) × 3	Maternal	No	Normal	4

Abbreviation: NA, not available.

**Table 3:** Maternal characteristics of amniocentesis of the 15q11.2 CNV groups and the normal array group.

Characteristics	15q11.2 BP1–BP2 Microdeletion (*n* = 10)	15q11.2 Microduplication (*n* = 11)	Normal Array (*n* = 897)	*p*-Value
Maternal age (years)	35.5 ± 3.0	35.3 ± 5.7	36.7 ± 3.9	0.34
Advanced maternal age	7 (70%)	9 (82%)	742 (83%)	0.51
Primipara	8 (80%)	6 (55%)	438 (49%)	0.08
Gestational age (weeks)	38.9 ± 1.3	38.1 ± 1.4	37.7 ± 2.3	0.22
Gender, male	3 (30%)	5 (46%)	485 (54%)	0.27
Follow-up period (months)	29.3 ± 21.7	28.8 ± 22.8	33.8 ± 17.1	0.49

Data presented as mean ± standard deviation or number (%).

**Table 4:** Inheritance pattern and ratio of the symptomatic parents of the 15q11.2 BP1–BP2 microdeletion and microduplication groups.

Inheritance Pattern	15q11.2 BP1–BP2 Microdeletion (*n* = 7) *	15q11.2 Microduplication (*n* = 11)	*p*-Value
De novo	0	2 (18%)	0.24
Paternal origin	4 (57%)	4 (36%)	0.40
Maternal origin	3 (43%)	5 (46%)	0.91
Symptomatic parents	4 (57%)	0	<0.05

Data presented as number (%) or mean ± standard deviation. * Only 7 of the 10 patients with 15q11.2 microdeletion with an inheritance pattern were analyzed.

**Table 5:** Perinatal outcomes between the 15q11.2 CNV groups and the normal array group.

Perinatal Outcome	15q11.2 BP1–BP2 Microdeletion (*n* = 10)	15q11.2 Microduplication (*n* = 9)	Normal Array (*n* = 897)	*p*-Value
Maternal outcome				
Cesarean section	4 (40%)	3 (33%)	419 (46.7%)	0.67
Postpartum hemorrhage	0	0	82 (9.1%)	0.39
Maternal comorbidity	0	0	150 (16.7%)	0.15
Neonatal outcome				
Preterm birth <37 weeks	1 (10%)	1 (11%)	156 (17.4%)	0.73
Low birth weight	2 (20%)	0	130 (14.5%)	0.41
Macrosomia	0	0	15 (1.7%)	0.85
Neonatal intensive care unit transfer	3 (30%) ^†^	0	60 (6.7%)	<0.05
1 min Apgar score <7	1 (10%) ^†^	1 (11%) ^‡^	15 (1.7%)	<0.05
5 min Apgar score <7	0	0	6 (0.7%)	0.94
Neonatal death	1 (10%) ^†^	0	5 (0.6%)	<0.05

Data presented as number (%). ^†^ Subgroup analysis detected a significant difference between the microdeletion group and the normal array group. ^‡^ Subgroup analysis detected a significant difference between the microduplication group and the normal array group.

**Table 6:** Early life outcomes of the children between the 15q11.2 CNV groups and the normal array group.

Early Life Outcome	15q11.2 BP1–BP2 Microdeletion (*n* = 10)	15q11.2 Microduplication (*n* = 9)	Normal Array (*n* = 897)	*p*-Value
Infantile death	2 (20%) ^†^	0	5 (0.6%)	<0.05
Symptomatic child	7 (70%) ^†,§^	2 (22%)	188 (21.0%)	<0.05
Developmental delay	5 (50%) ^†,§^	0	31 (3.5%)	<0.05
Growth delay	3 (30%) ^†^	0	19 (2.1%)	<0.05
Speech delay	2 (20%) ^†^	0	15 (1.7%)	<0.05
Motor delay	1 (10%) ^†^	0	9 (1.0%)	<0.05
Facial dysmorphism	1 (10%) ^†^	1 (11%) ^‡^	4 (0.4%)	<0.05
General behavioral problems	0	0	3 (0.3%)	0.97
Congenital heart disease ^¶^	3 (30%)	0	107/496 (21.6%)	0.23
Abnormal brain imaging ^¶¶^	1/5 (20%)	1/3 (33%)	67/501 (13.4)	0.55

Data presented as number (%). ^†^ Subgroup analysis detected a significance between the microdeletion group and the normal array group. ^‡^ Subgroup analysis detected a significance between the microduplication group and the normal array group. ^§^ Subgroup analysis detected a significance between the microdeletion group and the microduplication group. ^¶^ Only 496 neonates had echocardiography in the normal array group. ^¶¶^ Only 5 cases in the microdeletion group, 3 cases in the microduplication group, and 501 cases in the normal array group underwent brain imaging.

## Data Availability

All the data presented in this study are available on request from the corresponding author, without undue reservation, to any qualified researcher.
